# A mixed methods study of HIV-related services in buprenorphine treatment

**DOI:** 10.1186/s13011-017-0122-5

**Published:** 2017-08-16

**Authors:** Hannah K. Knudsen, Jennifer Cook, Michelle R. Lofwall, Sharon L. Walsh, Jamie L. Studts, Jennifer R. Havens

**Affiliations:** 1 Department of Behavioral Science and Center on Drug and Alcohol Research, University of Kentucky, 845 Angliana Ave, Room 204, Lexington, KY 40508 USA; 2 Department of Behavioral Science and Center on Drug and Alcohol Research, University of Kentucky, 845 Angliana Ave, Room 214, Lexington, KY 40508 USA; 3 Department of Behavioral Science and Center on Drug and Alcohol Research, University of Kentucky, 845 Angliana Ave, Room 203, Lexington, KY 40508 USA; 4 Department of Behavioral Science and Center on Drug and Alcohol Research, University of Kentucky, 845 Angliana Ave, Room 202, Lexington, KY 40508 USA; 5 Department of Behavioral Science, University of Kentucky, 1100 Veterans Drive, Medical Behavioral Science Building, Room 127, Lexington, KY 40536–0086 USA; 6 Department of Behavioral Science and Center on Drug and Alcohol Research, University of Kentucky, 845 Angliana Ave, Room 201, Lexington, KY 40508 USA

**Keywords:** Buprenorphine, Opioid use disorder treatment, HIV/AIDS testing, HIV prevention

## Abstract

**Background:**

Opioid use disorder (OUD) is a major risk factor in the acquisition and transmission of HIV. Clinical practice guidelines call for the integration of HIV services in OUD treatment. This mixed methods study describes the integration of HIV services in buprenorphine treatment and examines whether HIV services vary by prescribers’ medical specialty and across practice settings.

**Methods:**

Data were obtained via qualitative interviews with buprenorphine experts (*n* = 21) and mailed surveys from US buprenorphine prescribers (*n* = 1174). Survey measures asked about screening for HIV risk behaviors at intake, offering HIV education, recommending all new patients receive HIV testing, and availability of on-site HIV testing. Prescribers’ medical specialty, practice settings, caseload demographics, and physician demographics were measured. Multivariate models of HIV services were estimated, while accounting for the nesting of physicians within states.

**Results:**

Qualitative interviews revealed that physicians often use injection behaviors as the primary indicator for whether a patient should be tested for HIV. Interviews revealed that HIV-related services were often viewed as beyond the scope of practice among general psychiatrists. Surveys indicated that prescribers screened for an average of 3.2 of 5 HIV risk behaviors (SD = 1.6) at intake. About 62.0% of prescribers delivered HIV education to patients and 53.2% recommended HIV testing to all new patients, but only 32.3% offered on-site HIV testing. Addiction specialists and psychiatrists screened for significantly more HIV risk behaviors than physicians in other specialties. Addiction specialists and psychiatrists were significantly less likely than other physicians to offer on-site testing. Physicians in individual medical practice were significantly less likely to recommend HIV testing and to offer onsite testing than physicians in other settings.

**Conclusions:**

Buprenorphine treatment providers have not uniformly integrated HIV-related screening, education, and testing services for patients. Differences by medical specialty and practice setting suggest an opportunity for targeting efforts to increase implementation.

## Background

Opioid use disorder (OUD) is a major risk factor in the acquisition and transmission of HIV due to injection and high-risk sexual behaviors [1, 2]. Despite the strong links between OUD and HIV/AIDS, services for these two conditions have been fragmented in the United States, with HIV clinical care occurring in medical settings and OUD services located in clinics that are not embedded within mainstream medical institutions [3, 4]. This fragmentation has led to repeated calls for greater integration, such as co-location of buprenorphine-naloxone treatment and HIV-related services within the same setting [5, 6].

Such integration may yield numerous health-related benefits. By reducing opioid use, buprenorphine treatment reduces the risk of HIV acquisition through injection [2, 6–8]. Among HIV-positive patients, buprenorphine treatment reduces needle-sharing [9]. When buprenorphine is integrated into HIV care clinics, patients initiating buprenorphine treatment are more likely to receive antiretroviral therapy (ART) as well as report improvements in HIV-related medical outcomes [10] and quality of life [11].

While buprenorphine treatment itself is an important HIV prevention strategy, HIV testing and brief interventions to decrease risky sexual behaviors represent additional services that may yield public health benefits [2]. As noted in models for increasing the identification of individuals with HIV and linking them to care, such as the National Institute on Drug Abuse’s Seek-Test-Treat-Retain initiative [12], it is vitally important to connect individuals, particularly those at high risk HIV, to testing services [13, 14]. Rapid HIV testing only requires a non-intrusive oral swab that yields initial results in a brief period of time [15]. Rapid HIV tests that are marketed directly to consumers cost less than $50 [16], and in health care settings, per-patient costs have been estimated at $22–$46 per patient depending on the counseling protocol [17]. Furthermore, HIV testing is included among the preventive services that are covered under the Affordable Care Act [18]. Testing, subsequent linkage to ART for individuals with HIV, and ART adherence have benefits for the health of individuals, and by reducing the individual’s viral load, prevents the further spread of HIV [19].

In substance use disorder (SUD) treatment, the feasibility of implementing HIV testing has been demonstrated [20]. On-site delivery of HIV testing in SUD treatment results in more individuals receiving their test results than when individuals are referred to an off-site provider [21]. Integration of HIV testing into SUD treatment is particularly important because these patients may be not receive routine primary medical care [22]. Despite the health benefits of early identification and linkage to care, health services research has shown that HIV-related services have not been extensively adopted in these settings. In both licensed opioid treatment programs (OTPs) that primarily dispense methadone and counseling-based SUD treatment centers, the adoption of HIV testing and other services remains limited [23–28], despite the Centers of Disease Control and Prevention’s clinical practice guidelines that recommend testing in these and other health care settings [29].

Less is known about the diffusion of HIV-related services in buprenorphine treatment. Although some OTPs and SUD treatment centers have adopted buprenorphine [30], the majority of physicians delivering buprenorphine treatment do so via prescriptions in office-based settings outside OTPs and the specialty SUD treatment system [31]. Research has shown that the delivery of risk reduction counseling by buprenorphine providers is feasible [32]. However, a 2008 survey of providers participating in the Substance Abuse and Mental Health Services Administration’s Physician Clinical Support System-Buprenorphine (PCSS-B) found that counseling about sexual risk behaviors was unevenly implemented, and less than half of these physicians conducted HIV testing [33].

The present study explores the delivery of HIV-related services (i.e., risk assessment, education, and testing) in buprenorphine treatment. Through qualitative interviews with buprenorphine experts, we sought to understand the roles of buprenorphine-prescribing physicians in the delivery of HIV prevention and testing. Then, using survey data from a national sample of buprenorphine prescribers, we constructed multivariate models to examine the adoption of risk assessment practices, delivery of HIV education, recommending that all new patients be tested for HIV, and the availability of on-site HIV testing.

## Methods

### Study design

This mixed methods study integrated data from qualitative interviews with buprenorphine treatment experts and quantitative survey data collected from a large national sample of buprenorphine prescribers. As noted by Palinkas and colleagues [34], this type of mixed methods approach can be best described as “qual➔QUAN”, in which qualitative data collection preceded and informed the quantitative survey design. All research procedures were approved by the Institutional Review Board of the University of Kentucky.

### Qualitative procedures and participants

In the qualitative phase, we recruited buprenorphine-prescribing expert mentors within the SAMHSA-funded Physician Clinical Support System-Buprenorphine (PCSS-B; now the PCSS-MAT). Staff from the American Academy of Addiction Psychiatry provided the study team with a list of the 88 mentors in May 2013. A purposive sampling strategy was used to recruit mentors from 21 unique US states who reflected the gender, medical specialty, and practice setting distribution of the PCSS-B mentors. This sample size is consistent with the recommendations of Creswell [35] and Sandelowski [36] for achieving saturation in formative qualitative research with health care providers.

Sampled individuals (*n* = 33) were sent up to 4 email invitations about our study. A telephone interview was scheduled at a time convenient for the physician, and a description of the study and the rights of research subjects was emailed before the interview. Participants provided verbal informed consent, and received a check for $100 for participating. Between July 2013 and January 2014, 21 interviews were conducted (response rate = 63.6%) by a master’s level research assistant who had prior qualitative research experience and was trained in the interviewing protocol.

Participants were predominantly male (81.0%, *n* = 17) and white (95.2%; *n* = 20). One participant identified as Asian American (4.8%). In terms of medical specialty, 57.1% (*n* = 12) were addiction specialists (e.g., addiction medicine, addiction psychiatry), 23.8% (*n* = 5) were general psychiatrists, and 19.1% (*n* = 4) worked in other specialties (e.g., internal medicine, family medicine, obstetrics/gynecology). About 33.3% (*n* = 7) were affiliated with an academic medical center, 9.5% (*n* = 2) were affiliated with a Veterans Administration medical center, and 57.1% (*n* = 12) were not affiliated with either type of medical center.

While interviews largely focused on the practices constituting high-quality buprenorphine treatment, one section included questions specific to HIV services. Physicians were asked, “What is the role of buprenorphine-prescribing physicians in the delivery of HIV prevention, testing, and treatment?” All interviews were digitally recorded and professionally transcribed.

A qualitative description approach was employed to analyze the data [37], which is well-suited when describing participants’ perceptions and developing quantitative measures [38]. The lead author (HKK) initially used an inductive approach to identify major themes and then assigned descriptive codes to segments of the transcripts. These descriptive codes were collated into a codebook. A second coder (JC) then read the transcripts and employed the codebook to independently code each transcript. An iterative process was used to reach consensus on the selection of quoted passages from the coded transcripts of the themes represented in the data.

### Quantitative survey procedures

Quantitative survey data were collected from a national sample of civilian physicians who prescribe buprenorphine for the treatment of OUD. Civilian physicians were identified the Drug Enforcement Agency’s May 2014 Controlled Substances Act Registrants database (*n* = 24,506) Waivered physicians were randomly sampled within states to achieve a national sample reflecting the geographic distribution of waivered physicians across the US.

Telephone screening was used to determine study eligibility. The primary criterion for eligibility was current treatment of at least one patient with buprenorphine for OUD at the time of screening. Up to 10 attempts were made to gather the screening information, after which another randomly selected physician from his or her state replaced that physician.

The survey protocol was informed by Dillman’s [39] tailored design method and consisted of an advance notification letter, express-mailing (i.e., FedEx® or US Priority Mail) the survey packet, sending a postcard reminder, and calling physicians after 6 weeks of non-response and re-sending the survey. Participating physicians received an honorarium of $100 by mail. Surveys were mailed to 3553 physicians identified as eligible during screening, and 1174 participated in the survey (33.0% response rate) between July 2014 and January 2017. Survey data were entered into the REDCap (Research Electronic Data Capture) system [40], which is hosted by the University of Kentucky’s Center on Clinical and Translational Science. Participant characteristics appear in Table 1.

**Table 1:** Characteristics of buprenorphine-prescribing physicians and their practices reported as mean (standard deviation) or percentage (count)

	Mean (SD) or % (*N*)	95% CI	*N*
Medical Specialty			1149
Addiction specialty (medicine or psychiatry)	21.6% (248)	19.2–24.0	
Psychiatry (i.e., adult and/or child psychiatry with no mention of addiction)	27.2% (312)	24.6–29.7	
All others (e.g., family medicine, internal medicine, primary care, emergency medicine)	51.3% (589)	48.4–54.2	
Practice Settings			
Individual medical practice	50.8% (587)	47.9–53.7	1155
Group medical practice	35.2% (406)	32.4–37.9	1155
Veterans Administration medical center (VAMC)	4.6% (53)	3.4–5.8	1155
Hospital (non-VAMC)	13.2% (152)	11.2–15.1	1155
Opioid treatment program (OTP dispensing methadone)	6.2% (71)	4.8–7.5	1155
Non-OTP substance use disorder treatment program	13.9% (161)	11.9–15.9	1155
Caseload Characteristics			
Percentage of past-year patients with heroin use disorder (but not prescription opioids)	23.5 (22.4)	22.2–24.8	1135
Percentage of past-year patients with prescription opioid use disorder (but not heroin)	54.5 (27.3)	52.9–56.1	1134
Percentage of past-year patients with co-occurring heroin and prescription opioid use disorder	22.8 (19.9)	21.6–23.9	1133
Physician Characteristics			
Age	55.5 (11.4)	54.8–56.1	1160
Female	22.9% (267)	20.5–25.3	1165
Race and ethnicity			1148
White	76.5% (878)	74.0–78.9	
Asian American	12.5% (144)	10.6–14.5	
African American/Black	4.7% (54)	3.5–5.9	
Hispanic/Latino	4.4% (50)	3.2–5.5	
All others	1.9% (22)	1.1–2.7	
Waivered to treat up to 100 patients	57.8% (678)	54.9–60.6	1174

*Note.* Percentages may not sum to 100% due to rounding

### Survey measures

Survey measures asked physicians about HIV risk assessment, education, and testing. First, physicians indicated (1 = yes, 0 = no) whether all new patients were asked about: (1) frequency of injection drug use; (2) sharing of syringes, (3) sharing of non-syringe drug paraphernalia, (4) number of sexual partners, and (5) frequency of unprotected sexual intercourse. The rationale for including these practices is that common HIV risk assessment instruments, such as the Risk Behavior Assessment [41] and the HIV Risk Questionnaire [42], focus on these behaviors. These items were summed into an index of HIV risk assessment practices that ranged from 0 to 5. A dichotomous measure asked physicians whether they delivered HIV education to their patients (1 = yes, 0 = no). Additional descriptive items asked about the extent (0 = no extent, 5 = very great extent) to which HIV educational efforts emphasized how HIV is transmitted, the importance of not sharing syringes and other drug paraphernalia, development of safer sex practices, rehearsing correct condom use, and communicating with partners about safer sex practices; physicians who reported not delivering HIV education were coded as “0” on these descriptive items. These measures were adapted from prior studies of HIV services in specialty SUD treatment [23, 43]. Dichotomous items asked physicians whether they recommended HIV testing to all new patients (1 = yes, 0 = no) and whether HIV testing was conducted on-site at their office (1 = yes, 0 = no).

Medical specialty and practice setting were two key independent variables of interest based on our qualitative findings. The survey asked physicians about their specialty or area of medical practice using an open-ended format. Trained research staff then coded these responses into three mutually exclusive groups: addiction specialists (addiction medicine or addiction psychiatry), psychiatrists (with no mention of addiction), and other prescribers from all other specialties.

Physicians were asked six dichotomous items whether they prescribed buprenorphine in individual medical practice, group medical practice, a Veterans Administration medical center (VAMC), a hospital (non-VAMC), an opioid treatment program (OTP, i.e., methadone program that also offers buprenorphine treatment), and/or a non-OTP SUD treatment program. Prescribers could indicate more than one setting, so these variables were treated as separate dichotomous variables.

To measure caseload characteristics, physicians indicated the percentage of their patients in the past year who had heroin use disorder (but not prescription opioid use disorder), the percentage who had both prescription opioid and heroin use disorder, and the percentage with prescription opioid use disorder (but not heroin use disorder). Because these three variables sum to 100% and are interrelated (an increase of one necessitates a decrease in the others), the multivariate models excluded the percentage of patients with co-occurring prescription opioid and heroin use disorder.

Physician characteristics including age in years, gender (1 = female, 0 = male), and race/ethnicity (with white as the reference category, Asian, and all others in the multivariate models) were measured. Information regarding whether the physician was waivered to treat up to 30 (=0) or 100 patients (=1) was extracted from the DEA’s May 2014 CSA database.

Finally, two state-level variables were incorporated into the model. Because our survey was fielded during the era of health reform, we incorporated a measure of state-level approaches to implementing the Medicaid expansion and state-based health insurance exchanges under the Affordable Care Act (ACA) of 2010. This federal legislation, which was implemented during the presidency of Barack Obama, sought to reduce the number of uninsured Americans and improve population health. The ACA required all Americans to obtain health insurance (called the “individual mandate”) and contained provisions to support access to health insurance, such as expanding Medicaid to include adults with incomes of less than 138% of the federal poverty line [44] and combinations of tax credits and subsidies for individuals purchasing coverage via state-based or federal insurance exchanges [45]. Our measure of ACA implementation represents a three-category typology based on information published by the Henry J. Kaiser Family Foundation [46, 47]. We categorized ACA-supportive states as those that expanded Medicaid and implemented a state-based health insurance exchange (15 states and the District of Columbia). ACA-hybrid states chose to either expand Medicaid or establish a state-based exchange, but did not implement both policies (11 states); 10 of these states implemented the Medicaid expansion. ACA-resistant states included states that chose to not expand Medicaid and did not establish a state-based exchange (24 states). A second state-level measure drew upon data on the number of individuals living with diagnosed HIV infection per 100,000 state residents in 2014 [48]. States below the mean (i.e., 299 individuals living with HIV/AIDS per 100,000 residents) were categorized as low prevalence states (=1; 32 states) and those at or above the mean were categorized as high prevalence states (=0; 18 states and District of Columbia).

### Quantitative data management and analysis

Descriptive statistics were calculated for all study variables. To avoid the bias that results from complete case analysis [49], multiple imputation by chained equations was implemented using “mi impute chained” in *Stata 13.1* as a method to address missing survey data [50]. Rates of missing data ranged from 0.8% for gender to 4.0% for the index of HIV risk assessment practices*.* Our specification of “mi impute chained” included the index of HIV risk assessment, the dichotomous measure of delivering HIV education, the measure of recommending HIV testing to new patients, availability of on-site HIV testing, and all of the independent variables, with each variable imputed based on the appropriate link function (e.g., logistic regression if dichotomous, Poisson regression if a count, etc.). Thirty datasets were generated from the imputation procedure, and these datasets were used for the estimation of mixed effects regression models for each of the dependent variables of interest (i.e., “melogit” for dichotomous dependent variables and “mepoisson” for the index of HIV risk assessment). Each of these models accounted for the nesting of physicians within states and incorporated the two state-level measures as fixed effects.

## Results

### Qualitative findings regarding buprenorphine prescribers as significant providers of HIV-related services

Almost all of the physicians in the qualitative sample indicated that buprenorphine prescribers should have a significant role in provision of HIV (and hepatitis C; HCV) services. One of the primary ways that buprenorphine prescribers talked about involvement was in relation to HIV testing. Some physicians indicated their support for testing all patients regardless of specific risk factors. For example, one physician stated:

“I don’t think it’s compulsory, but I think that is a standard of care. I mean, I think you need to do some lab work when you bring patients in. You know HIV and HCV. Absolutely I think it’s below standard of care if you’re not assessing any patient that is a substance abuser, particularly if they’re intravenous. But I do it on all of them.” (Male, Addiction Specialist)

Others also discussed the importance of assessing HIV risk and delivering counseling related to risky behaviors. Many physicians noted the importance of determining whether or not the patient injected drugs. Others indicated the need to assess for other risk factors, as noted by the following physician: “Obviously every Suboxone® doctor should be asking about HIV risk factors, not just, you know, IV drug use but also unprotected sex” (Male, Addiction Specialist). Some also discussed the importance of providing risk reduction counseling to their patients, like the following physician who discussed risk reduction counseling for individuals who tested negative for HIV:

“And then the role would be if it’s negative to try to reinforce the fact you’re negative; please don’t engage in risky behavior, please don’t inject any longer. I might say to a patient, you know if you do relapse, if that should happen, if you could just use it intranasally and not inject, that would be much better than injecting.” (Male, General Medicine)

These physicians acknowledged the importance of addressing risk factors and providing counseling services to reduce the risk of their patients contracting HIV.

Physicians also described the significance of their role in providing services for HIV-positive patients. Many physicians highlighted the importance of referring HIV-positive patients to HIV care, such as the following physician who stated, “and if they’re positive, well then they have to be referred for appropriate treatment, which some HIV waivered physicians can do” (Male, General Medicine). Thus, these physicians highlight the importance of ensuring their patients are referred to an HIV specialist to receive the treatment they need to meet all of their medical needs.

### Qualitative findings about focusing HIV services only on “high risk” patients

Although almost all physicians discussed the importance of HIV services for patients who use drugs, some physicians only delivered such services to individuals who reported injection drug use. For example, one physician stated: “But I would say that when you take your initial history from the opioid addicted patient sitting there, that certainly if somebody has been an injection drug user, that that would be something that, you know, they should just have a preliminary HIV and hepatitis C screening to see if they’re negative or not” (Male, Addiction Specialist). This physician highlighted how he would definitely recommend an HIV test for those who injected drugs but did not indicate whether HIV testing would be important for all patients. Another doctor was more specific, indicating that he only recommended testing for patients who have injected drugs:

“Oh, I only test people for HIV and hepatitis if they’re IV drug users. And the majority of most of my patients are prescription drug users who don’t, you know, don’t use needles. So I do test everybody who’s an IV drug user. I may ask them if they’ve shared or reused the needle and, if they’ve never done that, I’m less likely (Male, Addiction Specialist).”

These data suggested that some buprenorphine physicians evaluated HIV risk solely based on injection use. In some cases, the physician acknowledged that not testing each patient did not align with recommendations of the CDC; as one physician noted, “Well I guess I think that the CDC, I think, has recommended that like everyone has an HIV test… Although I don’t really follow that rule but I think that that is something they did recommend” (Male, General Medicine).

A minority of physicians indicated they did not perceive their patient population to be at high risk for HIV, and thus, did not deliver any HIV testing or prevention services. For example, one physician had difficulty addressing the question about HIV service delivery due to his perception of low injection drug use among his patients: “That’s a kind of more of a philosophical question. I don’t deal very much with positive HIV folk; I haven’t seen too many… Because first they don’t, well in [this state] there’s much less IV use of opiates” (Male, Psychiatrist). The physician’s focus on his entire state rather than specific individuals within his practice led him to conclude that HIV testing and prevention were not needed despite CDC recommendations regarding universal testing. Similarly, another physician cited a low rate of needle-sharing in his population as a reason not to deliver HIV services: “Most people these days that I treat are negative…Because of needle exchanges or just being able to go to the drug store and buy sterile needles” (Male, General Medicine).

### Qualitative findings on HIV services by physician specialty and practice setting

Another major theme that emerged was how the provision of HIV risk assessment, education, and testing to patients varied by the medical specialty and practice setting of the physician. Many physicians indicated that specialties like family medicine and primary care were better suited to deliver HIV-related services than physicians who do not provide primary care services, such as psychiatrists. One physician discussed how he believed many non-primary care specialties avoid HIV risk assessment and HIV testing due to the detailed medical history and laboratory work involved: “Unfortunately I don’t think it happens very regularly much because, and not that one needs to be a primary care, in general medicine, family practice, to go into this, but I think a part of every evaluation needs to include a complete history, physical exam, and appropriate laboratory screening” (Male, Addiction Specialist). Other physicians indicated they were not typically involved in HIV service delivery because they assumed primary care doctors filled this role. One psychiatrist perceived that she should focus only on how buprenorphine may interact with other drugs each patient may take, by stating, “You know, I’m a psychiatrist. I think a primary care doctor is more involved, but certainly psychiatrists, you know, we do need to know drug/drug interactions and that sort of thing” (Female, Psychiatrist). Another physician described how HIV testing was the responsibility of primary care providers, as illustrated in the following interaction between the interviewer (I) and respondent (R):

“I: What would you say is the role of buprenorphine prescribing physicians in the delivery of HIV prevention, testing and treatment?

R: None.

I: Okay. Why?

R: Yeah, we do some, we do some but, well okay, that’s not a true statement. So for the ones who were in, you know like, first line settings where they are methadone clinic type settings. You know where you’re taking people who are untreated and then obviously HIV testing is important in that setting. Okay. For the docs who have the more established patients and the higher functioning patients, … a lot of those patients have the, have, already have primary care, I think that’s the primary care’s responsibility.” (Female, Addiction Specialist)

### National survey results regarding availability of HIV-related services

Table 2 presents descriptive data regarding the availability of HIV-related services in buprenorphine treatment. Nearly all respondents indicated that they asked all new patients about injection drug use, and most physicians asked about syringe sharing. Fewer physicians asked all new patients about sharing other types of paraphernalia, the number of sexual partners, and frequency of unprotected intercourse. The majority of physicians reported that they delivered HIV education to patients. The means for the specific elements of HIV education were at or below the midpoint for these scales. The means for education focused on sexual risks were even lower and about half the magnitude of the means for education focused on drug-related risks. About half of the sample recommended HIV testing to all new patients, yet only about one-third of physicians offered on-site HIV testing.

**Table 2:** Characteristics of HIV-related services in buprenorphine treatment reported as mean (standard deviation) or percentage (count)

	Mean (SD) or % (*N*)	95% CI	*N*
Intake practices^a^			
All new patients are asked about frequency of injection drug use	92.4% (1055)	90.8–93.9	1142
All new patients are asked about sharing of syringes	82.9% (944)	80.7–85.1	1139
All new patients are asked about sharing of non-syringe drug paraphernalia (e.g., straws, cottons, cookers)	53.2% (604)	50.3–56.1	1136
All new patients are asked about number of sexual partners	48.0% (548)	45.1–50.9	1141
All new patients are asked about frequency of unprotected sexual intercourse	48.5% (554)	45.6–51.4	1142
Count of intake practices	3.2 (1.6)	3.1–3.3	1127
HIV education			
Physician delivers HIV/AIDS education to his/her patients^*a*^	62.0% (709)	59.2–64.8	1144
Patient education emphasis on how HIV/AIDS is transmitted^*b*^	2.0 (1.9)	1.9–2.2	1136
Patient education emphasis on the importance of not sharing syringes^*b*^	2.5 (2.2)	2.4–2.6	1137
Patient education emphasis on the importance of not sharing other drug paraphernalia^*b*^	2.1 (2.1)	1.9–2.2	1136
Patient education emphasis on development of safer sex practices^*b*^	2.1 (2.0)	2.0–2.2	1132
Patient education emphasis on skill rehearsal of correct condom use^*b*^	0.7 (1.4)	0.7–0.8	1335
Patient education emphasis on practicing partner communication and negotiation skills about safer sex practices^*b*^	1.0 (1.5)	1.0–1.1	1133
Mean scale of patient education	1.7 (1.6)	1.7–1.8	1127
HIV testing^a^			
Recommend that all new patients be tested for HIV/AIDS	53.2% (610)	50.3–56.1	1146
On-site HIV testing (rapid or non-rapid)	32.3% (370)	29.5–35.0	1147

*Notes.* ^a^Dichotomous measures (1 = yes, 0 = no). ^b^Likert responses (0 = no extent to 5 = very great extent)

### Multivariate models of HIV-related services

Table 3 presents the results from four mixed effects regression models of the adoption of risk assessment practices, delivery of HIV education, recommending that all new patients be tested for HIV, and the availability of on-site HIV testing. Differences by medical specialty varied depending on the type of HIV-related service. Addiction specialists and psychiatrists both reported assessing a greater number of the five types of HIV risk behaviors than physicians in other specialties. Addiction specialists and psychiatrists were significantly more likely to deliver HIV education to patients. However, these two types of specialists were significantly less likely than physicians in other specialties to offer on-site HIV testing. Furthermore, psychiatrists were significantly less likely to recommend HIV testing to all new patients, relative to physicians from non-addiction/non-psychiatry specialties.

**Table 3:** Multivariate models of HIV services in buprenorphine treatment

	Model 1 Sum of intake practices			Model 2 Delivers HIV education			Model 3 Recommends HIV testing to all new patients			Model 4 On-site HIV testing		
	IRR	95% CI	*p*	OR	95% CI	*p*	OR	95% CI	*p*	OR	95% CI	*p*
Medical Specialty												
Addiction specialty	1.145	1.049–1.250	0.003	1.437	1.011–2.043	0.043	1.321	0.925–1.885	0.125	0.580	0.396–0.848	0.005
Psychiatry	1.109	1.020–1.206	0.016	1.213	0.887–1.659	0.226	0.518	0.374–0.720	<.001	0.270	0.183–0.397	<.001
All others (reference)	1.000			1.000			1.000			1.000		
Individual medical practice	1.031	0.937–1.133	0.535	1.317	0.871–1.989	0.191	0.574	0.387–0.850	0.006	0.459	0.304–0.694	<.001
Group medical practice	0.996	0.905–1.095	0.932	1.358	0.893–2.065	0.153	0.883	0.591–1.319	0.543	0.935	0.617–1.417	0.751
Veterans Administration medical center (VAMC)	1.077	0.915–1.268	0.374	3.659	1.568–8.539	0.003	3.757	1.691–8.348	0.001	5.099	2.496–10.418	<.001
Hospital that is not a VAMC	1.056	0.955–1.167	0.288	1.808	1.177–2.776	0.007	1.366	0.910–2.051	0.133	1.598	1.038–2.460	0.033
Opioid treatment program (OTP dispensing methadone)	1.101	0.961–1.261	0.167	2.925	1.496–5.717	0.002	1.491	0.816–2.725	0.194	1.447	0.799–2.617	0.222
Non-OTP substance use disorder treatment program	0.982	0.886–1.089	0.730	1.169	0.756–1.807	0.482	0.749	0.492–1.141	0.178	0.503	0.313–0.806	0.004
Percentage of past-year patients with heroin use disorder	0.999	0.997–1.001	0.212	0.995	0.987–1.003	0.227	1.002	0.993–1.010	0.709	1.006	0.997–1.014	0.198
Percentage of past-year patients with prescription opioid use disorder	0.996	0.994–0.998	<.001	0.990	0.983–0.997	0.004	0.987	0.980–0.994	<.001	0.998	0.990–1.005	0.525
Age	1.003	1.000–1.007	0.034	0.992	0.980–1.004	0.199	1.010	0.997–1.022	0.120	0.978	0.965–0.991	0.001
Female	1.010	0.931–1.096	0.812	1.140	0.831–1.563	0.416	1.512	1.090–2.096	0.013	1.362	0.974–1.906	0.071
Race and ethnicity												
White (reference)	1.000			1.000			1.000			1.000		
Asian American	0.987	0.888–1.096	0.802	0.853	0.577–1.260	0.424	1.463	0.975–2.195	0.066	0.714	0.450–1.131	0.151
African American, Hispanic/Latino, and all others	1.032	0.927–1.150	0.562	0.862	0.572–1.300	0.479	1.014	0.665–1.547	0.948	0.790	0.499–1.251	0.315
100-patient waiver	0.915	0.854–0.979	0.010	0.823	0.634–1.068	0.143	0.800	0.610–1.048	0.105	0.860	0.646–1.145	0.303
Affordable Care Act (ACA) state typology												
ACA-resistant state (reference)	1.000			1.000			1.000			1.000		
ACA-hybrid state	1.044	0.937–1.164	0.432	1.333	0.897–1.980	0.154	1.170	0.703–1.949	0.546	1.014	0.598–1.722	0.958
ACA-supportive state	1.034	0.947–1.129	0.450	1.331	0.978–1.812	0.069	1.604	1.053–2.444	0.028	1.150	0.744–1.777	0.530
State has below-average prevalence of HIV	0.999	0.923–1.082	0.986	0.913	0.678–1.229	0.549	1.090	0.743–1.600	0.660	1.289	0.875–1.899	0.200
*Constant*	3.167	2.467–4.064	<.001	2.769	1.026–7.473	0.044	1.453	0.527–4.008	0.470	3.196	1.072–9.528	0.037
*Random Effects Parameters:* Variance (constant)	0.002	0.000–0.048		0.019	0.000–8.584		0.155	0.051–0.475		0.136	0.041–0.456	

*Notes.* Pooled estimates are presented from 30 imputed datasets, with each dataset containing 1174 physicians. The same 30 imputed data sets were used for estimating each of the four models. Mixed effects Poisson regression was used for the model of intake practices, while the other three models were estimated using mixed effects logistic regression. The incidence rate ratios (IRR) and odds ratios (OR) were tested using *t* statistics with average degrees of freedom of 15,106.99 (Model 1), 29,407.23 (Model 2), 44,194.57 (Model 3), and 47,684.53 (Model 4). F-statistics were F(18, 184,977.8) = 3.77 (*p* < .001), F(18, 341,410.0) = 3.46 (*p* < .001), F(18, 367,008.2) = 5.82 (*p* < .001), and F(18, 453,841.0) = 7.31 (*p* < .001) for Models 1–4, respectively

Practice settings were associated with the odds that physicians delivered HIV education, recommended testing, and offered on-site HIV testing. Physicians delivering buprenorphine in individual medical practice were less likely to recommend HIV testing and less likely to offer on-site HIV testing, relative to physicians in other settings. However, physicians working in VAMCs, relative to those not delivering buprenorphine in VAMCs, were significantly more likely to deliver HIV education, recommend all new patients be tested for HIV, and to offer on-site HIV testing. Delivering buprenorphine in a non-VAMC hospital was positively correlated with the odds of delivering HIV education and offering on-site HIV testing. The only HIV service associated with delivering buprenorphine in an OTP was HIV education, and this association was positive in direction. Physicians practicing in non-OTP specialty SUD programs were significantly less likely than those in other settings to report on-site HIV testing. Delivering buprenorphine in a group medical practice was not associated with any of the four HIV services. Notably, none of the practice settings were correlated with the index of risk assessment intake practices.

Caseload characteristics were correlated with some HIV-related services. The percentage of OUD patients in treatment because of prescription opioids (but not heroin) was negatively associated with three of the four HIV-related services. Specifically, physicians who treated a higher percentage of patients for prescription opioids reported using significantly fewer HIV risk assessment practices and were significantly less likely to deliver HIV education. Furthermore, physicians with larger caseloads of prescription opioid patients were significantly less likely to recommend HIV testing to all new patients. However, the percentage of patients who were in treatment because of heroin was not associated with any of the four HIV-related services.

Physicians’ characteristics were correlated with delivery of some HIV-related services. Age was positively correlated with the number of risk assessment practices during intake, but negatively correlated with the likelihood that the physician offered on-site HIV testing. Female physicians were more likely than male physicians to recommend HIV testing to all new patients. There were no differences by race/ethnicity. The only difference by the type of buprenorphine waiver was that physicians with the 100-patient waiver had adopted fewer of the HIV risk assessment practices than physicians with the 30-patient waiver.

Finally, there was little evidence that the two state characteristics were associated with HIV services. The only significant difference was that physicians in ACA-supportive states were more likely than those in ACA-resistant states to recommend HIV testing to all new patients. The state-level prevalence of people living with HIV/AIDS was not associated with any of the HIV-related services.

## Discussion

The elevated risk of HIV acquisition and transmission for individuals with OUD suggests the need for integrating HIV services within buprenorphine treatment settings. The CDC’s guidelines recommend the integration of HIV testing in all medical settings [29]. With the advent of rapid HIV tests that do not require phlebotomy facilities, adoption and implementation should be feasible in a range of settings. However, this mixed methods study found that the availability of specific HIV-related services was quite variable in buprenorphine treatment. Availability was correlated with medical specialty, practice setting, and the percentage of patients in treatment because of prescription opioids.

Physicians’ focus on assessing HIV risk via injection-related risk behaviors was borne out in both our qualitative data and our national survey. In the qualitative interviews, buprenorphine experts often described the need to ask patients about injection behaviors. Our survey data was consistent with this finding, as the vast majority of buprenorphine prescribers reported asking new patients about injection and syringe sharing. Yet, there was evidence in both the qualitative interviews and survey data that some physicians assumed that individuals seeking buprenorphine treatment because of prescription opioids were at limited risk of acquiring HIV. In the multivariate models, physicians treating greater percentages of such patients reported using fewer risk assessment practices, were less likely to deliver HIV education, and were less likely to recommend HIV testing. Epidemiological studies have shown that many individuals who misuse prescription opioids do inject these substances [51–53], so perceptions of limited HIV risk among those who use prescription opioids may result in missed opportunities for intervention. Focusing on individuals who inject opioids may be targeting those at highest risk, but individuals who do not inject still face risks if they engage in risky sexual behaviors.

Both the qualitative interviews and survey data revealed considerably less emphasis on sexual risk behaviors in physicians’ assessment of HIV risk. Such findings are not altogether unexpected, given that medical providers across specialties are often reticent to discuss sexual behaviors [54]. Even in HIV care clinics when HIV-positive patients disclose high-risk sexual behaviors, providers are often reluctant to deliver risk reduction counseling to patients [55, 56]. Nonetheless, limited implementation of assessment of risky sexual behaviors and sexual risk reduction counseling represents a missed opportunity. Epidemiological research has shown that sexual transmission of HIV is common among individuals who use drugs, whether they inject or not [57], underscoring the need to follow CDC recommendations for HIV testing. Reductions of risky sexual behaviors may reduce the incidence of new cases of HIV [58], and counseling by medical providers is one intervention that may target these behaviors.

Models for improving the identification of individuals with HIV and linking them to care, such as Seek-Test-Treat-Retain [12], begin with efforts to encourage individuals at high-risk of HIV to be tested [13, 14]. Our survey data showed that only about half of buprenorphine physicians recommended HIV testing to all new patients, and only one-third offered on-site HIV testing. This uneven implementation of HIV testing was consistent with an earlier survey conducted by Edelman et al. [33]. Both our qualitative and quantitative findings suggest that many buprenorphine prescribers see HIV testing as outside their scope of practice because of their medical specialties and delivery settings. Buprenorphine experts drew boundaries between psychiatry and general medical care, and our survey results demonstrated significant differences in adoption of HIV testing between psychiatrists and those in non-addiction/non-psychiatry specialties. Furthermore, addiction specialists were less likely to have adopted on-site HIV testing than physicians from other medical specialties. The significantly lower adoption of testing by those in solo practice also corroborated our qualitative findings. Given that ordering laboratory tests are within the scope of practice for all medical specialties, it is somewhat surprising that some physicians viewed ordering an HIV test as outside their own scope of practice.

It is important to note that limited implementation of HIV testing is not unique to physicians offering buprenorphine treatment. A survey of primary care physicians in the state of New York found only about 40% of physicians routinely implement HIV testing [59]. Limited adoption of HIV testing in specialty SUD treatment settings has been repeatedly documented [23–28, 60]. Nonetheless, the elevated prevalence of HIV among individuals who use drugs suggests that lack of adoption of HIV testing may reduce the likelihood that patients learn their status, which is a critically important first step in linking individuals who test positive to HIV care. Thus, we recommend that the buprenorphine waiver training courses review these recommended HIV services along with models of their effective adoption in order to ensure that new providers are being adequately educated and trained.

There are a number of limitations inherent in the study design. Both the qualitative and quantitative data collection were cross-sectional in their design, so causal claims and inferences cannot be made based on these findings. The measures of HIV-related services have not, to our knowledge, ever been validated against objective measures, such as health records or patients’ reports of service receipt. We only considered a limited range of independent variables. Other variables, such as HIV stigma, may serve as barriers to the adoption of HIV-related services; future research should consider this possibility. In addition, the qualitative phase focused on individuals who served as mentors to others interesting in implementing buprenorphine treatment; they likely constitute a unique subset of providers. Their perspectives on the delivery of HIV services may differ from individuals who are less experienced or who have not committed to mentoring others. However, consistency between our qualitative results and the statistical models do suggest that the themes identified may have some resonance with the broader field of buprenorphine prescribers.

An additional limitation is that, at the time of the study, only physicians were permitted to prescribe buprenorphine and for most of the study period, physicians were limited to no more than 100 concurrent patients. Recent policy changes [61] will soon allow nurse practitioners and physician assistants to prescribe buprenorphine, which may allow buprenorphine to diffuse to additional settings. The current study on HIV-related services cannot speak to the types of HIV services that may be available once nurse practitioners and physician assistants are able to deliver buprenorphine treatment. These professions typically are oriented toward preventive services, of which HIV testing is one element. Adding information about HIV education and testing to the new training requirements for this potentially large group of future providers may be an important consideration. In addition, physicians can now apply to treat up to 250 patients. Given that we found that higher volume providers (i.e., those with the 100-patient waiver) were not more likely to offer HIV-related services, it may suggest that extra efforts, such as continuing medical education, may be needed to prompt the adoption of such services by high volume buprenorphine providers with large caseloads of individuals with opioid use disorder.

A substantial limitation of the survey data was a limited response rate. Large-scale surveys of physicians often face this challenge [62, 63]. We are unable to estimate the impact of non-response, although we will be able to compare respondents and non-respondents once we complete our planned 12-month follow-up survey. Although higher response rates are often assumed to be superior to lower response rates, the broader literature on survey design has revealed increases in response rates are not as influential as many assume. Research has shown that response rates have minimal impact on point estimates [64] as well as correlations between variables [65]. Nonetheless, the extent to which these findings generalize to those who did not participate in the survey is unknown.

## Conclusion

This mixed methods study revealed ongoing challenges to the full integration of HIV-related services into the buprenorphine treatment system in the US. The limited adoption of HIV prevention and HIV testing suggests that future research should focus on identifying barriers to providing HIV testing as well as testing implementation strategies to expand the delivery of these HIV-related services by buprenorphine providers. Implementation strategies often combine a variety of efforts, such as strategic planning, training, identification of funding, and restructuring work processes, to increase the use of a given intervention [66]. The growing field of implementation science [67–70] can be drawn upon to identify potential combinations of strategies that may prove fruitful in increasing the integration of HIV services in buprenorphine treatment. Additional dissemination of the CDC guidelines may raise awareness about the importance of HIV testing and risk reduction counseling [71]. Training may be important to reduce provider discomfort in offering HIV testing [72], to identify procedures for follow-up when patients test positive for HIV [73], and to inform providers about state regulations related to testing [20]. Implementation efforts in large health systems, such as the Veterans Administration, have shown that rates of HIV testing increased after system change initiatives that included social marketing of HIV testing to providers, training for clinic staff, clinical reminders within the electronic health record, and publicity campaigns within clinic waiting rooms [74]. By documenting the limited adoption of HIV testing among addiction specialists and psychiatrists as well as those in solo practice, these findings suggest that buprenorphine providers may be important groups to target in future implementation research. Buprenorphine providers may serve an even more significant role in reducing the spread of HIV if greater implementation of HIV-related services can be achieved.
